# Relationship between vitamin D levels and pediatric celiac disease: a systematic review and meta-analysis

**DOI:** 10.1186/s12887-024-04688-0

**Published:** 2024-03-16

**Authors:** Yanhong Sun, Qingxue Zhou, Dandan Tian, Jianming Zhou, Shilei Dong

**Affiliations:** 1 Department of Clinical Laboratory, National Clinical Research Center for Child Health, , National Children’s Regional Medical Center, Children’s Hospital, Zhejiang University School of Medicine, Hangzhou, 310052 China; 2 Department of Clinical Laboratory, Hangzhou Women’s Hospital, Hangzhou, 310008 China; 3 Department of Clinical Laboratory, Zhejiang Hospital, Hangzhou, 310013 China

**Keywords:** 25-hydroxyvitamin D_3_, Meta-analysis, Pediatric celiac disease, Vitamin D

## Abstract

The relationship between Vitamin D levels and pediatric celiac disease (CD) remains controversial. In this study, we conducted a systematic review and meta-analysis to examine the relationship between Vitamin D and pediatric CD. Methods: We screened relevant studies from PubMed, EMBASE, and Web of Science published in English from January 1, 2000, to August 1, 2023. The included studies were assessed according to the STROBE checklist. Heterogeneity was quantified by Cochran’s Q test and the I^2^ statistic. Publication bias was estimated by Begg’s test and Egger’s test. Meta‐regression was used to detect potential sources of heterogeneity. Results: A total of 26 studies were included in the meta-analysis. Nineteen articles compared 25(OH)D3 levels between CD patients and control groups, average 25-hydroxyvitamin D_3_ [25(OH)D_3_ or calcidiol], and 1,25-dihydroxyvitamin D_3_ [1,25(OH)_2_D_3_ or calcitriol] levels, as the main forms of Vitamin D, there was a significant difference in CD patients and healthy controls (weighted mean difference (WMD) = − 5.77, 95% confidence interval (CI) = [− 10.86, − 0.69] nmol/L). Meanwhile, eleven articles reported the numbers of patients and controls with Vitamin D deficiency, there was a significant difference in the incidence of 25(OH)D_3_ deficiency between CD patients and healthy controls (odds ratio 2.20, 95% CI= [1.19, 4.08]). Nine articles reported changes in 25(OH)D_3_ levels before and after administering a GFD in patients with CD, the result of this study revealed the increase of 25(OH)D_3_ levels in CD patients after a gluten-free diet (GFD) (WMD = − 6.74, 95% CI = [− 9.78, − 3.70] nmol/L). Conclusions: Vitamin D levels in pediatric CD patients were lower than in healthy controls, and 25(OH)D_3_ deficiency was more prevalent in CD patients. We found that 25(OH)D_3_ levels were elevated in CD patients after GFD, which is consistent with previous research. Further well-designed, longitudinal, prospective cohort studies focusing on the role of Vitamin D in the pathogenesis of CD are therefore needed.

## Introduction

Celiac disease (CD) is an immune-mediated systemic disorder caused by intolerance to gluten found in barley and rye [1]. CD affects the small intestine in genetically susceptible individuals [2]. Improvements in the diagnosis and awareness of the disease have greatly increased the apparent incidence of CD [3], with a 2–4-fold increase in the number of confirmed CD patients in Europe and the United States during the last two decades [4, 5]. Research has shown that the incidence of CD to be increasing by 7.5% per year over the past several decades [5]. Of 65,957 screened children, 891 had CD, with a prevalence of CD is 0.9%, which was significantly greater in children than adults (0.5%) according to the global prevalence of CD [6]. The prevalence of CD varied from 0.3% in Iran to 0.7% in Israel between various Asian countries [7]. CD mainly affects the small intestine, however, the clinical manifestations are extensive, with both intestinal and parenteral symptoms [8]. The intestinal and extra-intestinal clinical symptoms of CD vary, but the most prominent symptom is proximal intestinal malabsorption, and chronic diarrhea, which can appear over weeks or months [1, 9]. The extra-intestinal symptoms include iron deficiency anemia, faltering growth, weight loss, failure to thrive, delayed puberty, and mouth ulcers [9, 10]. Although the incidence of pediatric CD is increasing, its pathogenesis remains unclear. CD generally occurs in genetically susceptible individuals who respond to unknown environmental factors with an immune response that is subsequently triggered by the intake of gluten [11]. Environmental factors, such as the duration of gluten exposure, play important roles in the development of CD [12]. However, CD occurs only in about 1% of the population, suggesting that other environmental factors besides gluten are probably important, such as Vitamin D, infant feeding practices, delivery method, the season of birth, elective cesarean section, intestinal microbiome, time of gluten introduction, acute viral gastrointestinal infection, and micronutrient deficiency [13–18]. Vitamin D levels may also be associated with CD, and Yavuz et al. revealed significantly reduced levels of Vitamin D in pediatric patients with newly diagnosed CD [19]. The main treatment for CD remains adherence to a lifelong GFD, which requires significant education, motivation, and follow-up of the patient, with improvement and resolution of symptoms usually occurring within days or weeks [20]. Two advanced clinical trials actively developing and testing pharmacological approaches to treat CD, include AT-1001 (Larazotide acetate, which aims to close the villi’s tight junctions) and IMGX-003 (Latiglutenase; formerly known as ALV003, acts as a gluten endopeptidase that degrades gluten before being absorbed in the small intestine) therapies [21].

Vitamin D is a fat-soluble vitamin that exerts its biological effects by binding to Vitamin D receptors [22], which are distributed in various tissues and cells throughout the human body, and subsequently participates in numerous biological processes, including immunity, metabolism, and inflammation [23, 24]. Vitamin D, as an immune modulator, is known to regulate immune response and maybe implicated in disease pathogenesis or susceptibility of CD. Components of the immune system, such as B-lymphocytes, T-lymphocytes, and dendritic cells, are influenced by the regulatory effects of Vitamin D and expressed Vitamin D receptor (VDR), which is involved in the biological activity of 1,25(OH)_2_D_3_, and these cells also have the capability of locally synthesizing active 1,25(OH)_2_D_3_ [25]. Increasing evidence also suggests that Vitamin D deficiency increases the risk and worsens the outcome of extraskeletal diseases such as cancer, irritable bowel syndrome, and inflammatory bowel disease [26].

GFD remains the only effective treatment for CD [27]. Strict adherence to a GFD over a year has been shown to lead to partial healing of the duodenal mucosa along with the resolution of gastrointestinal and extraintestinal manifestations as well as complications such as malabsorption and osteoporosis while having similar results in mucosal structural recovery, reduction in intestinal mucosal inflammation, antibody concentrations, and symptom improvement [28–30]. Cross-contamination of GFD foods has emerged as a threat to chronic low-dose gluten exposure in CD patients, daily gluten intake below a specific threshold should be determined [31]. Several studies established a safe threshold of daily gluten intake, 100 mg gliadin/day (= about 200 mg of gluten or 2–5 g wheat flour) displayed minimal morphometric changes in the jejunal histology [32]. Gluten-free foods are less available and cost more, while in CD with GFD, symptoms can relieved and quality of life can be significantly improved [33, 34]. Beyond patients with CD, GFD is also recognized in the treatment of gluten ataxia, dermatitis herpetiformis, cognitive impairment, inflammatory bowel disease and irritable bowel syndrome, dermatitis herpetiformis, and non-celiac gluten sensitivity [27]. Adverse events of GFD may worsen the gut microbiota while having nutritional deficiencies in iron, calcium, and fiber, also include the negative social and psychological impacts that many GFD adherents experience [35, 36].

However, the relationship between Vitamin D and pediatric CD presents new challenges. For example, Ahlawat et al. found no significant difference in Vitamin D levels between patients with newly diagnosed CD and controls [37]. We therefore conducted a systematic review and meta-analysis to quantify the relationship between Vitamin D and pediatric CD, with the aim of providing new clues to the cause of pediatric CD.

## Methods

### Data selection

We searched for relevant articles published in English from January 1, 2000, to July 1, 2023, in the PubMed, EMBASE, and Web of Science databases. The following search terms were used: ((Coeliac disease) OR (gluten-induced enteropathy) OR (gluten-sensitive enteropathy) OR (Celiac disease) OR (Celiac sprue)) AND ((Vitamin D) OR (25(OH)D_3_) OR (Cholecalciferol) OR (25-Hydroxyvitamin D) OR (Hydroxycholecalciferols) OR (Ergocalciferols) OR (Dihydrotachysterol)) AND ((children) OR (adolescent) OR (pediatric)). The inclusion criteria were: (1) published as full English research articles; (2) pediatric CD; (3) unified definition and diagnosis of CD; and (4) supporting data for Vitamin D. Articles that did not meet the above criteria and duplicate publications were excluded. The main form of Vitamin D is 25(OH)D_3_ level, which is usually tested as a measure of Vitamin D levels. A 25(OH)D_3_ unit is defined as 1 ng/mL = 2.5 nmol/L, and levels of 25(OH)D_3_ were categorized as normal (≥ 70 nmol/L), insufficient (< 70 nmol/L), and deficient (< 50 nmol/L) [38].

Articles for selection were screened independently by two authors, who also screened the identified full articles. In the case of disagreement, the articles were evaluated by a third author to reach a final agreement.

### Article assessment

Risk of bias and quality assessment were assessed according to the STROBE checklist for the included studies [39]. The meta-analysis was also conducted in accordance with the Preferred Reporting Items for Systematic Reviews and Meta-Analyses (PRISMA) statement [40].

### Data extraction

Data were extracted to Numbers (Apple Distribution International, Seattle, USA) for statistical analysis. The following data were obtained from the included studies: basic characteristics including author, published year, country, detection method of 25(OH)D_3_, and diagnostic criteria of CD; 25(OH)D_3_ levels (mean ± standard deviation (SD)) in CD patients and controls; numbers of CD patients and controls; and treatment method of CD. Finally, all data were double-checked by two authors.

### Statistical analysis

In this study, we analyzed weighted mean difference (WMD) after combining mean and SD values for 25(OH)D_3_ levels. Odds ratios (ORs) and 95% confidence intervals (CIs) were used to calculate the incidences of 25(OH)D_3_ deficiency in CD patients and healthy controls. Statistical heterogeneity was assessed by Cochran’s Q test and the I^2^ statistic. For heterogeneous results, publication bias was estimated by Begg’s test and Egger’s test (*P* > 0.5, there is no publication bias). Pooled estimates were obtained using a fixed-effects model (Mantel and Haenszel, M-H) if I^2^ ≤ 50% and *P* > 0.1, or a random‐effects model (M‐H heterology) if I^2^ > 50% and *P* ≤ 0.1. Potential sources of heterogeneity were detected by meta-regression analysis (Monte Carlo permutation test) to enhance the credibility of the results. All analyses were carried out using STATA (Version 15.1, StataCorp., College Station, TX, USA) and Review Manager (Version 5.3, The Nordic Cochrane Centre, Rigshospitalet, Denmark).

## Results

### Basic characteristics

**Fig. 1:**
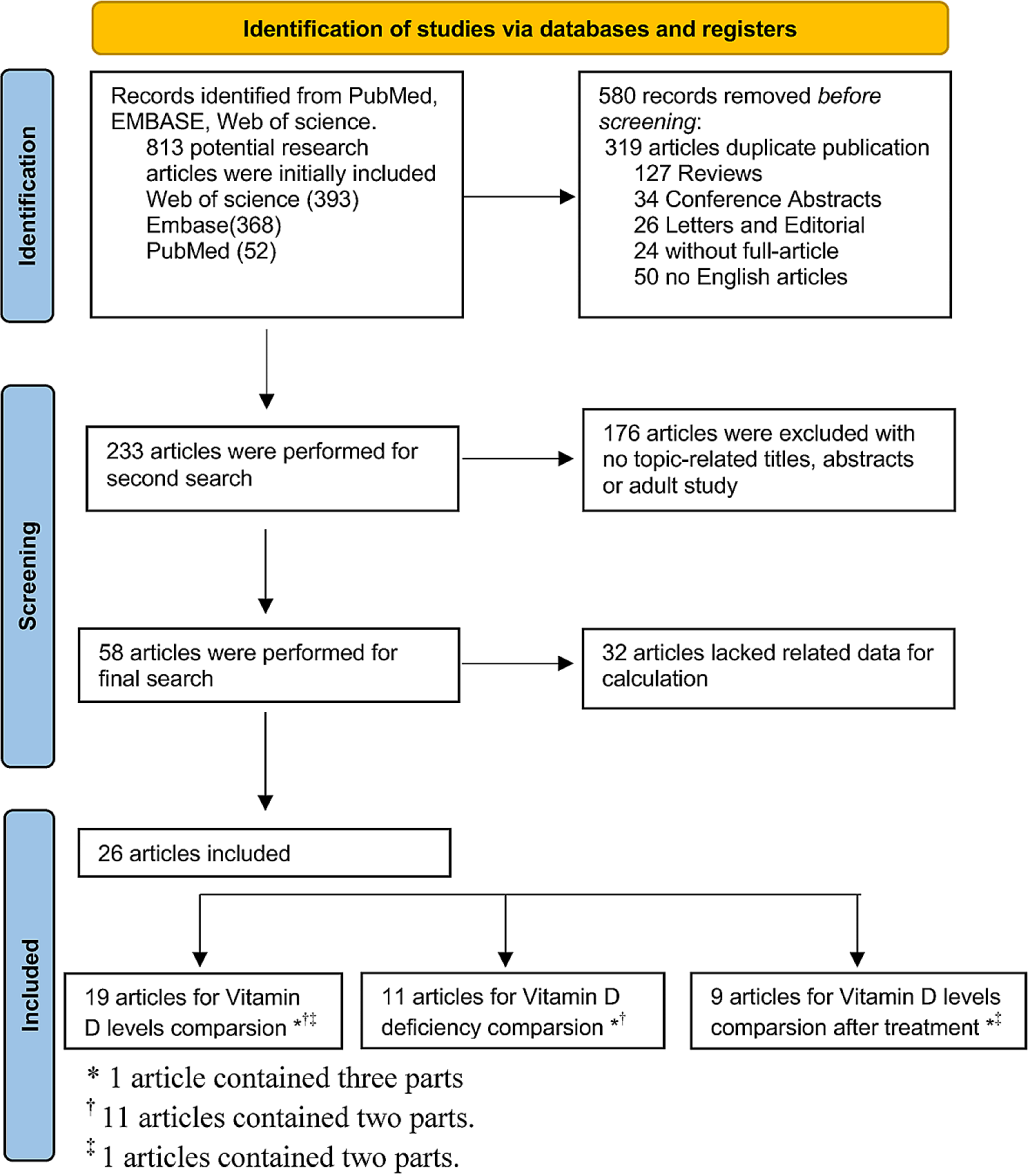
Flowchart of study-selection process

A total of 813 potential unique references were searched, of which 26 studies [19, 37, 41–65] met the inclusion criteria. A flowchart describing the study-selection process is shown in Fig. 1. The 26 studies included 3,120 subjects, comprising 1,495 CD patients and 1,607 non-CD participants. Most of the subjects were from the Middle East, Europe, and North America. CD was mainly diagnosed in the duodenal mucosa and by positive serological markers of disease. Most studies analyzed 25(OH)D_3_ by chemiluminescent immunoassay. Nineteen articles compared 25(OH)D_3_ levels between CD patients and control groups [19, 37, 41–44, 46, 49–52, 54–57, 59, 60, 66, 67], of which eleven found lower 25(OH)D_3_ levels in CD patients compared with controls [41, 42, 44, 49, 52, 55, 57, 59, 60, 66, 67]. Eleven articles reported the numbers of patients and controls with Vitamin D deficiency [19, 37, 42, 46, 49, 50, 52, 54, 57, 59, 67], and eight showed that 25(OH)D_3_ deficiency was more prevalent in CD patients [19, 42, 49, 50, 52, 57, 59, 67]. In addition, nine articles reported changes in 25(OH)D_3_ levels before and after administering a GFD in patients with CD [41, 47, 48, 52, 56, 61–65], all concluded a significant increase in Vitamin D levels after a GFD treatment [41, 56, 61–64]. The meta-analysis was conducted using a random-effect model or fixed effect model.

### Comparison of 25(OH)D_3_ levels in CD patients and healthy controls

**Fig. 2:**
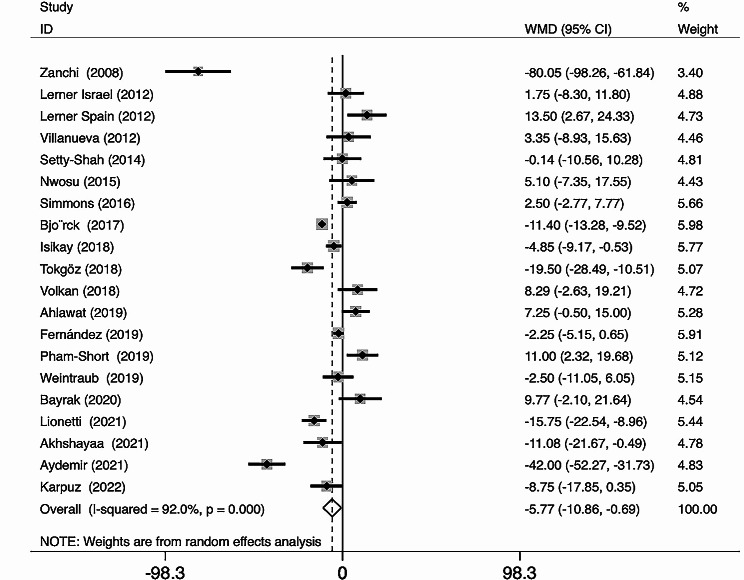
Forest plot showing significant difference in average 25(OH)D_3_ levels between pediatric CD patients and controls (WMD = − 5.77, 95% CI = [− 10.86, − 0.69] nmol/L)

**Fig. 3:**
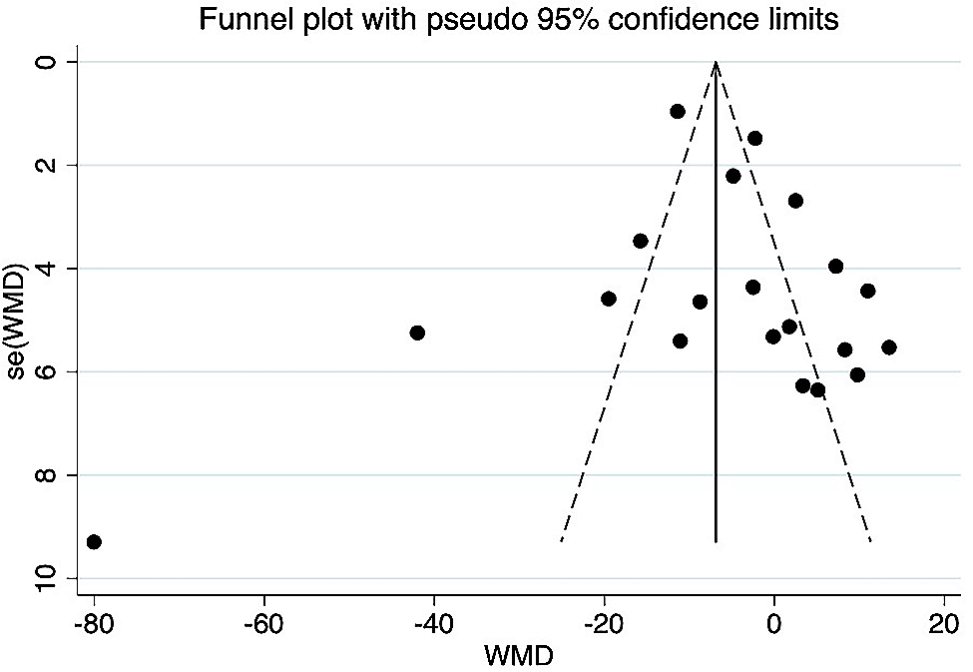
Funnel plot of the meta-analysis

The included studies, comprising 1,495 CD patients and 1,607 healthy controls, are listed in Table 1. The mean 25(OH)D_3_ levels were 57.39 nmol/L in CD patients and 64.08 nmol/L in healthy controls. The average 25(OH)D_3_ level in CD patients was 6.7 nmol/L lower than that in controls, and the difference was significant (WMD = − 5.77 95% CI = [− 10.86, − 0.69] nmol/L) (Fig. 2). As shown in Fig. 3, the funnel plot was used to identify the presence of the publication bias preliminary. Funnel plot is a simple scatter plot that reflects estimates of the intervention effects of a single study with a given sample size or accuracy, a useful tool for meta-analysis, which can be combined with relevant statistical tests to check for reporting bias in systematic reviews preliminary [68]. Funnel plot asymmetry cannot be equated with publication bias, because it has several other possible causes, such as heterogeneity, reporting bias, and chance may all lead to asymmetry or other shapes in funnel plots [69]. Funnel plots can help guide the choice of meta-analysis method. Random effects meta-analyses weight studies relatively more equally than fixed effect analyses by incorporating the between study variance into the denominator of each weight [70]. The asymmetry of the distribution of studies in the plot may be due to selection bias, publication bias citation bias, or multiple publication bias. The P values of Begg’s test and Egger’s test are 0.95 and 0.54, respectively, both > 0.5, indicating no publication bias. There was no abnormal sensitivity analysis, and meta-regression found no heterogeneity in terms of location, race, or publication year.

**Table 1:** Basic characteristics of studies of Vitamin D levels in patients with CD and healthy controls

Author	Year	Country	CD			Control		
			*N*	Mean	SD	*N*	Mean	SD
Zanchi	2008	Italy	54	56.53	28.78	60	136.58	65.28
Lerner	2012	Israel	51	64	24.5	56	62.25	28.5
Lerner	2012	Spain	59	75.75	30.75	56	62.25	28.5
Villanueva	2012	USA	24	68.95	24.78	50	65.5	26.13
Setty-Shah	2014	USA	40	65.11	24.04	49	65.25	26.03
Nwosu	2015	USA	25	70.6	25.7	49	65.4	26.1
Simmons	2016	USA	123	85	22.5	129	82.5	20
Bjo ¨rck	2017	Sweden	66	65.7	7.04	140	77.1	4.81
Işıkay	2018	Turkey	226	30.98	32	268	35.83	9.5
Tokgöz	2018	Turkey	52	49.5	19.75	50	69	26
Volkan	2018	Turkey	72	47.79	23.57	30	39.5	26.44
Ahlawat	2019	USA	38	66	20	82	58.75	20.5
Fernández	2019	Spain	67	70	8.22	66	72.25	8.81
Pham-Short	2019	Australid	42	76	22	40	65	18
Weintraub	2019	Israel	47	65	20.37	66	67.5	25.93
Bayrak	2020	Turkey	228	57.1	86.18	135	47.33	23.53
Lionetti	2021	Italy	131	63.25	20	131	79	34.25
Akhshayaa	2021	India	60	44.35	23	60	55.43	34.96
Aydemir	2021	Turkey	36	33.5	17.13	36	75.5	26.38
Karpuz	2022	Turkey	54	47.5	22.5	54	56.25	26.53

CD: celiac disease; SD: standard deviation

**Fig. 4:**
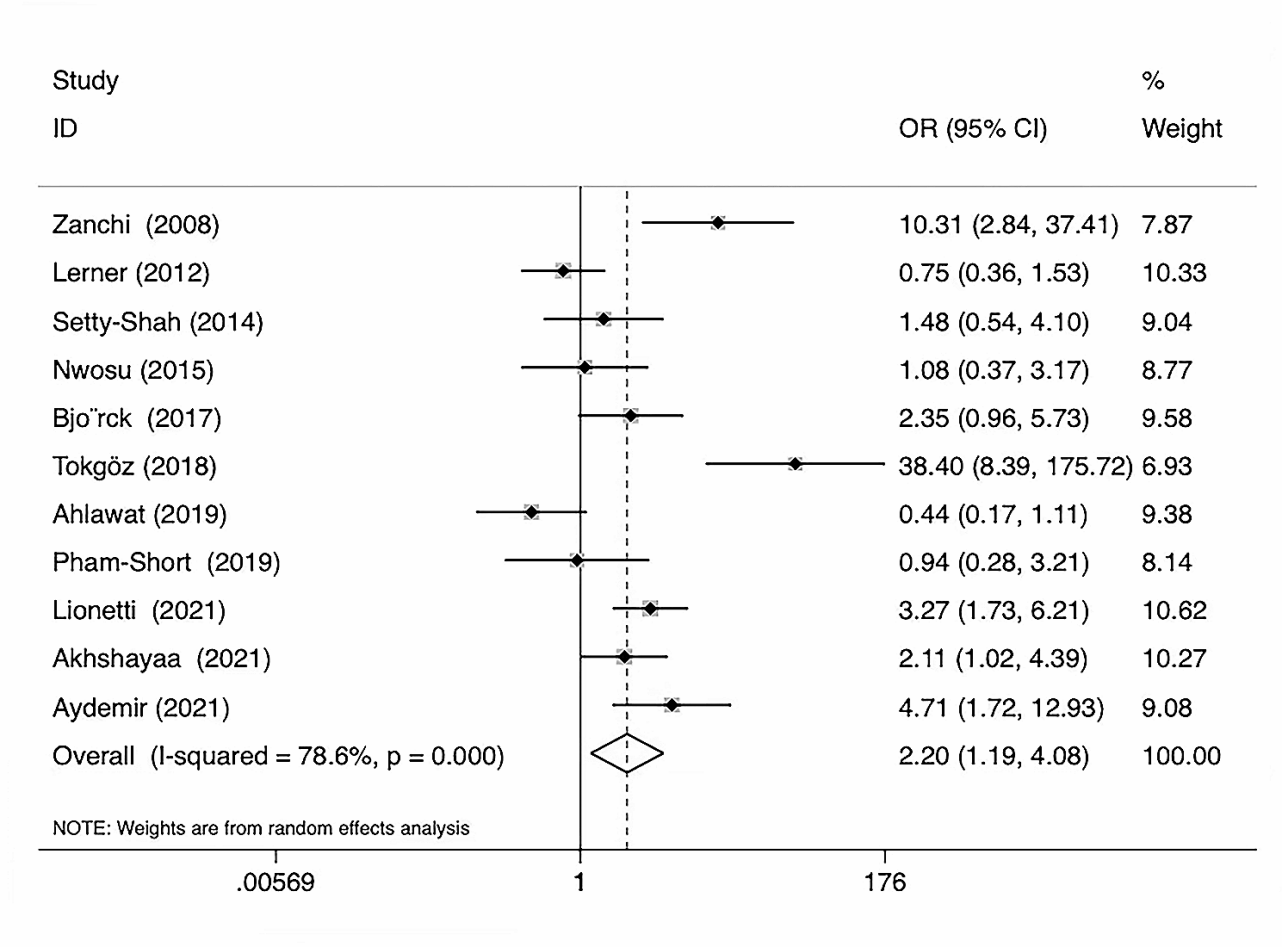
Forest plot showing significant difference in the incidence of 25(OH)D_3_ deficiency between patients with CD and healthy controls (OR = 2.20, 95% CI = [1.19, 4.08])

The pooled data from four included studies showed 25(OH)D_3_ deficiency in of 33.96% patients with CD and 18.60% of healthy controls. The meta-analysis found a significant difference in the incidence of 25(OH)D_3_ deficiency between CD patients and healthy controls (OR = 2.20, 95% CI = [1.19, 4.08]) (Fig. 4).

### Changes in 25(OH)D_3_ levels after CD treatment

**Fig. 5:**
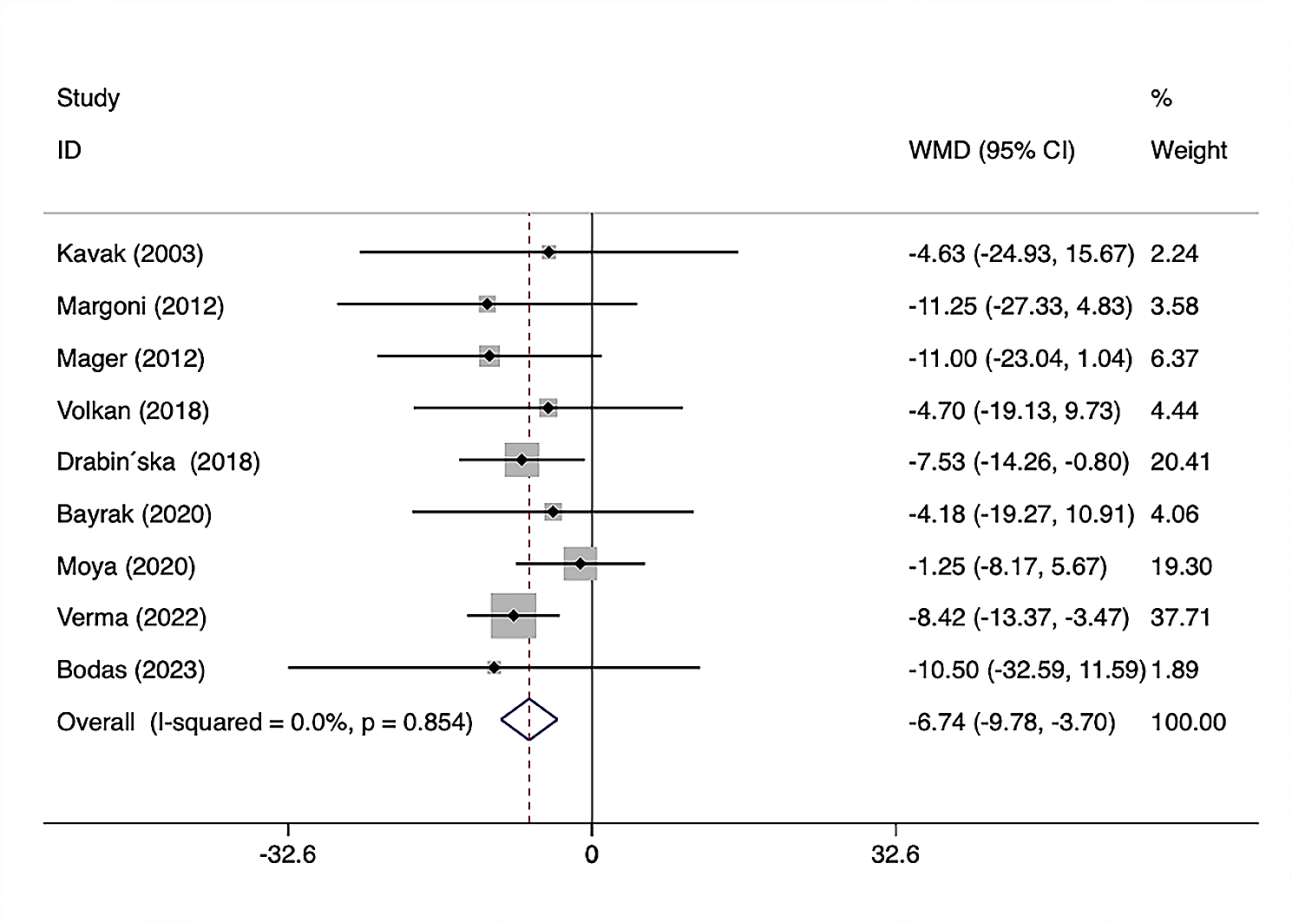
Forest plot showing no significant difference in 25(OH)D_3_ levels between CD patients pre-treatment and post-treatment with GFD (WMD = − 6.74, 95% CI = [− 9.78, − 3.70] nmol/L)

We examined changes in 25(OH)D_3_ levels in patients with CD before and after a GFD treatment in nine articles with relevant information (Table 2). All articles concluded the increase in Vitamin D levels after a GFD treatment, comprising 624 CD patients and 532 healthy controls. The results produced by STATA software, WMD were analyzed after combining mean and SD values for 25(OH)D_3_ levels, and 95% CIs were used to calculate the changes of 25(OH)D_3_ before and after a GFD treatment in CD patients. Overall WMD = − 6.74, 95% CI = [− 9.78, − 3.70] nmol/L, the diamond shape did not pass through the origin, indicating a significant increase in 25(OH)D_3_ levels in CD patients before and after a GFD treatment. (Fig. 5).

**Table 2:** Basic characteristics of studies of Vitamin D levels in patients with CD before and after GFD

Author	Year	Country	CD pre-treatment			CD post-treatment				
			*N*	Mean	SD	*N*	Mean	SD		
Kavak	2003	Turkey	34	75.25	39.75	28	79.88	41.25		
Margoni	2012	Greece	45	62	26.25	36	73.25	43.25		
Mager	2012	Canada	34	77	22	31	88	27		
Volkan	2018	Turkey	26	44.7	22.98	21	49.4	26.68		
Drabin ´ska	2018	Poland	16	47.75	7.09	16	55.28	11.76		
Bayrak	2020	Turkey	228	57.1	85.93	192	61.28	71.83		
Moya	2020	USA	155	75.25	30	122	76.5	28.5		
Verma	2022	India	60	37.13	13.48	60	45.55	14.18		
Bodas	2023	Spain	26	65.43	31.78	26	75.93	47.88		

CD: celiac disease; GFD: gluten free diet; SD: standard deviation

## Discussion

The results of previous studies regarding differences in Vitamin D levels between pediatric patients with CD and healthy controls have been inconsistent. The results of the current meta-analysis suggested that Vitamin D levels in pediatric CD patients were lower than healthy individuals, the 25(OH)D_3_ deficiency was more prevalent in CD patients. However, the Vitamin D levels in CD patients did not change before and after GFD. Overall, these results indicate the need for further research into the role of Vitamin D in the pathogenesis of CD.

The current study demonstrates a relationship between pediatric CD and Vitamin D, Vitamin D may be related to the incidence of CD. Gastrointestinal infections may be related to CD [71], and rotavirus infection in children has been reported to be a risk factor for the development of CD [72]. In addition, early infection in the neonatal period and childhood and antibiotic exposure have also been closely related to the occurrence of CD [73, 74]. Meanwhile, Vitamin D is involved in the process of resistance to infection. Vitamin D has been reported to induce the antibacterial peptide cathelicidin [75], which has in turn demonstrated direct antibacterial, antifungal, and antiviral properties [76]. The anti-infective properties of Vitamin D may thus be related to the onset of CD. Vitamin D levels may be influenced by three potential sources: nutrient sources, UVB-dependent endogenous production, and supplements. Most of the studies were from the Middle East, Europe, and North America. Some countries like the United States and Canada fortify staple products such as dairy products with Vitamin D. Thus, the individual Vitamin D dietary intake is highly dependent on nutritional habits, and the country’s fortification strategy [25]. However, a global perspective review found that 6 to 47% of Vitamin D intake may come from dietary supplements [77, 78]. Thus, Vitamin D status in the absence of supplementation is largely determined by endogenous Vitamin D production, which is also influenced by genetic determinants, latitude, season, skin pigmentation, and lifestyle [79, 80]. Because Vitamin D levels have been shown to be seasonally dependent, an individual’s 25(OH)D_3_ levels are lowest after winter and highest in late summer [81]. This factor should therefore be taken into account when interpreting individual Vitamin D levels.

Vitamin D can regulate both innate and adaptive immune system activity [82]. The risk of CD development is greater when patients with potentially associated Vitamin D hypovitaminosis [83]. Vitamin D may have a key role in CD onset by two key factors: immune response regulation through action on dendritic cells and T-cells, and on intestinal permeability by regulating inflammatory cytokines and zonulin release pathway [84]. Vitamin D in the intestines can maintain gut homeostasis by synthesis 1α,25(OH)_2_D_3_ and VDR expression, especially since an optimal 1α,25(OH)_2_D_3_ status is vital, as it participates in regulatory activities regarding not only calcium absorption but also infection protection, epithelial barrier function preservation, and gut microbiota modulation [85]. Vitamin D in CD can also affect tight junctions, which are the major junctions responsible for intestinal mucosa barrier regulation [86]. Research showed 25(OH)D_3_ concentrations < 30 nmol/L and > 75 nmol/L during early infancy were associated with an increased risk of developing celiac disease autoimmunity in genetically at-risk children [87]. However, the conclusion on VD supplementation in patients with celiac disease is still unclear. Patients with Vitamin D deficiency (< 30 nmol/L) were given 60,000 IU of Vitamin D per week during the first 3 months of treatment, after which vitamin D was discontinued and they were advised to follow GFD, after 6 months, showed a significant increase in Vitamin D serum levels (from 23.63 ± 1.13 nmol/L to 33.83 ± 3.8 nmol/L), but no case reached normal VD values [64]. Vitamin D status could be affected by compliance with the gluten-free diet, poor absorption, and decreased intake [88]. However, regardless of Vitamin D levels at onset or during GFD, most experts recommend monitoring Vitamin D serum levels in all patients, especially when Vitamin D deficiency is recommended to correct with Vitamin D supplements [89]. More studies are warranted to evaluate the effect of strict dietary adherence to the GFD and its effect on Vitamin D supplements.

Previous studies have shown that vitamin D levels in the CD group are negatively correlated with symptom severity, which means the lower the vitamin D levels, the more severe the symptoms of CD patients [44]. Vitamin D deficiency is associated with reduced expression of the Vitamin D receptor and epithelial barrier proteins E-cadherin and claudin-2, which play an important role in children with CD in correlation with histological manifestations of disease severity [59]. These findings suggest that in CD patients, the structure of the paracellular pathway responsible for calcium absorption is disturbed and that Vitamin D deficiency exacerbates CD.

The proximal small intestine is the most commonly implicated intestine segment in CD, leading to disrupted absorption of some nutrients such as Vitamin D and the occurrence of diarrhea, further suggesting a possible relationship between Vitamin D deficiency and CD progression. Since the amount of those autoantibodies such as anti-endomysium and anti-transglutaminase is positively correlated to the degree of intestinal atrophy and the magnitude of the inflammatory infiltrate, the minor role played by Vitamin D malabsorption in CD is supported [90]. However, no deficiencies in other fat-soluble vitamins, such as vitamins K and A, have been found. The limited number of studies has led to inconsistent results. For example, Imam et al. observed that deficiencies of fat-soluble vitamins were uncommon in children with a new diagnosis of CD, suggesting that routine measurement of fat-soluble vitamin levels may not be necessary [91]. However, Vitamin D levels are known to be affected by many factors, such as diet and sun exposure, especially in children [22]. These issues may be affected by various factors during childhood. The Fok1 T-allele of Vitamin D receptor has an association with serum 25(OH)D_3_ deficiency in patients with CD, which plays a critical role in immunomodulation and maintaining barrier integrity [92]. The current meta-analysis shows an association between Vitamin D levels and the deficiency and CD, whether it is involved or not in the pathogenesis cannot be ruled out.

Significant difference in 25(OH)D_3_ levels between CD patients pre-treatment and post-treatment with GFD, research showed that oligofructose-enriched inulin added to the GFD essentially can improve Vitamin D and E status in pediatric CD patients [62]. Lu et al. observed CD had decreased serum 25(OH)D_3_ levels, which returned to normal after treatment [93]. Further studies of the effects of GFD in CD patients are needed to clarify the correlation. Given that GFD is a strict lifelong gluten-free therapy to maintain a healthy status, the nutrient intakes will change, calcium, magnesium, iron, and Vitamin D intakes were particularly insufficient in pediatric CD, it may expose CD patients to high fat and low essential micronutrient intakes, nutrition intake must be monitored to prevent the occurrence of diseases during treatment, such as cardiovascular or bone disorders [94].

Deficiency of Vitamin D related to nutrient malabsorption secondary to epithelial damage is frequent in untreated CD patients,

This study had several limitations. First, we did not perform subgroup analysis because the number of included studies was relatively small. Second, there were high heterogeneity and confounding factors in this meta-analysis, including age, sex, ethnicity, season, diet intake, and treatment. Compared to the normal controls, children with CD had a high intake of fiber, glycemic index, and glycemic load and lower intakes of folate [95]. However, not all the included studies provided adjusted values and we were therefore unable to pool the results by adjusting for these confounders. Finally, there was a lack of prospective studies and randomized controlled trials. Thus, although the study found a correlation between Vitamin D levels and CD, it lacks causal relationships, and we could only state that Vitamin D levels seem to differ between pediatric patients with CD and healthy controls. Further studies of the effects of Vitamin D supplementation in patients with CD could provide further evidence for any relationship. In addition, previous original studies did not adjust for potentially important confounders, such as body mass index, race, and dietary habits. Finally, although the existence of heterogeneity might bias the results, the current analysis found no major source of heterogeneity, suggesting that it was appropriate to carry out pooled analyses.

## Conclusion

Our study showed that 11 articles found that 25(OH)D_3_ levels were lower in CD patients compared to controls, 8 articles showed that 25(OH)D_3_ deficiency was more prevalent in CD patients, and 9 articles concluded that vitamin D levels increased significantly after a GFD treatment. In summary, Vitamin D levels were lower in pediatric patients with CD compared with healthy controls, Vitamin D deficiency was prevalent in pediatric CD patients, while Vitamin D levels increased after a GFD according to the result of meta-analysis, demonstrating that Vitamin D may play a critical role in pediatric CD. Further prospective studies are therefore needed to clarify the association between Vitamin D and CD, including randomized controlled trials of the effects of Vitamin D supplementation in patients with CD.

## Data Availability

All data generated or analysed during this study are included in this published article.
